# RNA-Seq and UHPLC-Q-TOF/MS Based Lipidomics Study in *Lysiphlebia japonica*

**DOI:** 10.1038/s41598-018-26139-4

**Published:** 2018-05-17

**Authors:** Xueke Gao, Junyu Luo, Limin Lü, LiJuan Zhang, Shuai Zhang, Jinjie Cui

**Affiliations:** Institute of Cotton Research, Chinese Academy of Agricultural Sciences/State Key Laboratory of Cotton Biology, Anyang, Henan 455000 China

## Abstract

Lipids play an important role in energy storage, membrane structure stabilization and signaling. Parasitoids are excellent models to study lipidomics because a majority of them do not accumulate during their free-living life-stage. Studies on parasitoids have mostly focused on the changes in the lipids and gene transcripts in hosts and little attention has been devoted to lipidomics and transcriptomics changes in parasitoids. In this study, a relative quantitative analysis of lipids and their gene transcripts in 3-days-old *Lysiphlebia japonica* larva (3 days after spawning) and pupae were performed using liquid chromatography, mass spectrometry and RNA-seq. Thirty-three glycerolipids and 250 glycerophospholipids were identified in this study; all triglycerides and the vast majority of phospholipids accumulated in the pupal stage. This was accompanied by differentially regulated lipid uptake and remolding. Furthermore, our data showed that gene transcription was up-regulated in key nutrient metabolic pathways involved in lipid synthesis in 3-days-old larvae. Finally, our data suggests that larva and pupa of *L*. *japonica* may lack the ability for fatty acids synthesis. A comprehensive, quantitative, and expandable resource was provided for further studies of metabolic regulation and molecular mechanisms underlying parasitic response to hosts defense.

## Introduction

Lipids have diverse structure and composition^1,2^, enabling them to serve a variety of functions including as energy sources, structural components of cell membranes and organelles, and signaling molecules. In insects, lipids are grouped as internal or cuticular based on their organismal position^3^. Internal lipids are important for processes like oogenesis, metamorphosis, starvation, flight, and diapause^4^. Triacylglycerols (TAGs) serve as major energy sources for many insects during non-feeding periods and long distance flights^5^. Further, phospholipids (PLs), function as structural components of membranes^6^.

Parasitic wasps have developed a special developmental program when their larval growth and pupation occur as parasites of another insect species. *Lysiphlebia japonica*, the parasitoid wasp used in this study uses aphids as their preferred hosts. Numerous studies have demonstrated that some parasitoid species, such as *Nasonia vitripennis*, cannot synthesize lipids^7–12^.

Most key enzymes involved in the conversion of carbohydrates to triglycerides through pyruvate metabolism and the citrate cycle are functional in parasitoid wasps^13^. However, fatty acid synthase (FAS) is absent from the genome of the parasitic fungus *Malassezia globosa*^14^, and is therefore a prime candidate for studies on the lack of lipogenesis. A previous study showed that almost all genes involved in lipid synthesis pathways are up-regulated in *Aphis gossypii* following parasitic infection by *L*. *japonica*^15^. However, there are no data on the expression of lipid synthesis pathway genes in *L*. *japonica* itself.

Parasitic wasps are rapidly emerging as powerful model organisms to study lipid metabolism. Particular interest has been on understanding lipidome alterations during pupation. Until recently, it was not possible to address the genetic or nutritional influence on the lipidome. Mass spectrometry has become instrumental in identifying a vast number of different lipid species and is now recognized as a premier tool for lipidomics studies^16^.

In this study, we aimed to unravel the lipidomics and transcriptional profile during the developmental stages of the parasitic wasp, *L*. *japonica*, using mass spectrometry-based lipidomics that enabled us to simultaneously analyze changes in glycolipids and phospholipids. Transcriptome analysis by RNA sequencing was also performed to determine the molecular regulation of lipid metabolism genes under during the different developmental stages of *L*. *japonica*.

Our data revealed unexpected shifts in lipid accumulation during larval growth and pupa development. Genes associated with key nutrient metabolic pathways in lipid synthesis were analyzed, and results suggest that TAG formation plays an important role in lipid homeostasis. The ability of fatty acid synthesis in *L. japonica* was also examined. These comprehensive datasets constitute a valuable resource for future research on changes in *L*. *japonica* lipid composition and gene expression.

## Results

### UHPLC-Q-TOF/MS Analysis of Lipid Extracts in *L*. *japonica*

Samples were processed for chromatographic separation and mass spectrometry. Lipids identification was performed based on the existing literature and lipid map (http://www.lipidmaps.org/). From the original file, 2,056 and 3,757 peaks were detected in the positive (POS) and negative (NEG) modes. A total of 33 kinds of TAGs and 250 PLs **(**POS and NEG) were identified from the lipids extracts. Among these, the lipids exhibiting a significant increase or decrease were analyzed further.

**Figure 1:**
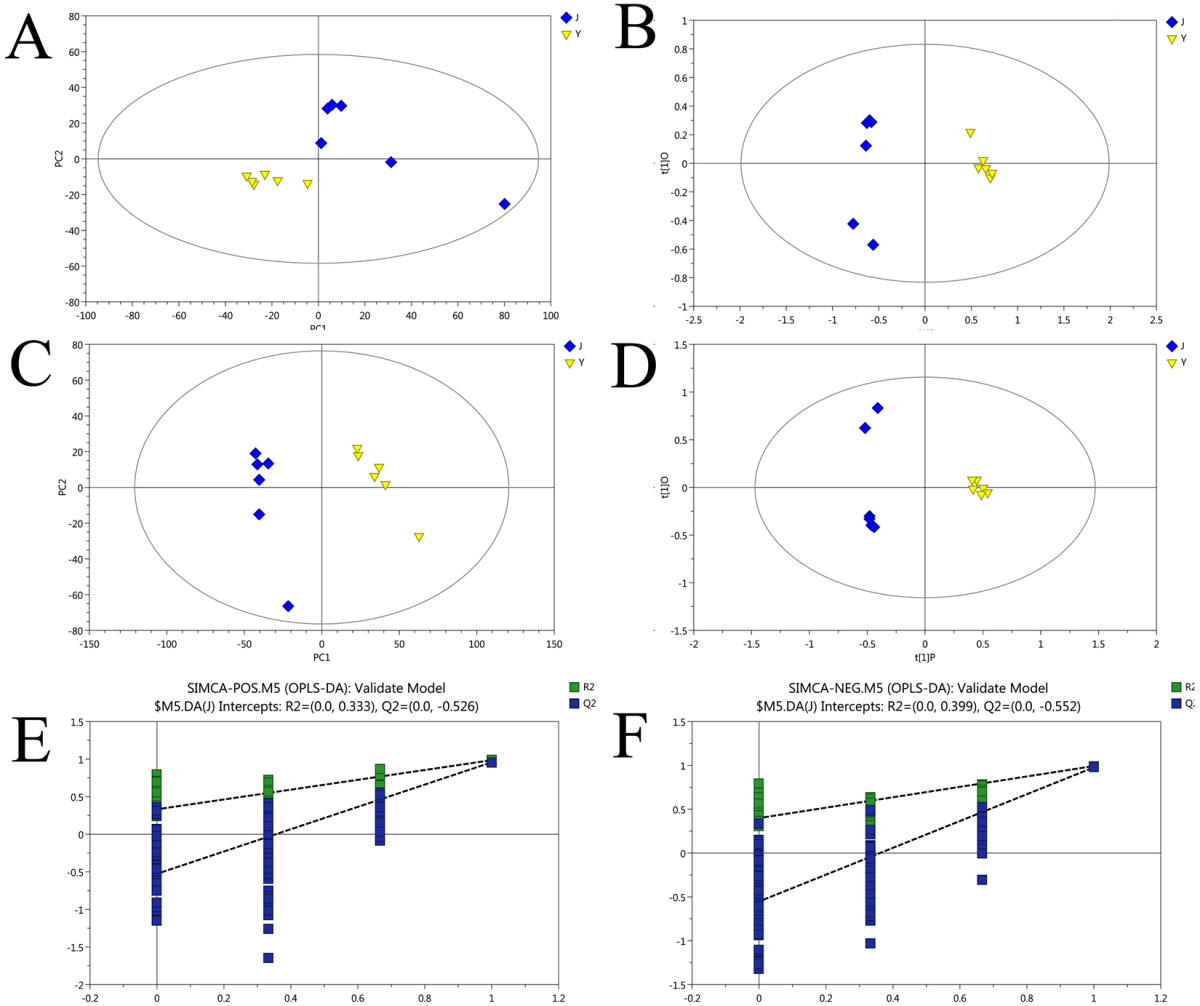
Score plot of PCA and OPLS-DA models of 3-days-old larvae (Y) and pupae (J) of *L*. *japonica* (POS and NEG). (**A**,**B**) Score plot of PCA model, obtained from Y and J (POS and NEG). (**C**,**D**) Score plot of OPLS-DA model, obtained from Y and J (POS and NEG). (**E**,**F**) Score plot of OPLS-DA model, obtained from Y and J (POS and NEG)^a^. The R2 factor estimates good fit, while the Q2 coefficient determines the predictive value of the created model referring to the percentage of correctly classified samples using cross-validation. ^a^Two hundred permutations were performed and the resulting R2 and Q2 values were plotted. (Green triangle): R2; (Blue square): Q2. The green and blue lines represent the regression lines for R2 and Q2, respectively.

In order to eliminate false positives and negatives, six independent pairwise comparisons were performed thus identifying statistically significant changes in lipid composition. In PCA, samples are classified based on a data matrix without prior information about class membership. PCA is a useful tool for classifying data, detecting outliers, and validating the stability and reproducibility of an analytical method. Graphical representation of the analytical PCA results is presented in Fig. 1(A,B), where lipids from groups J and Y are clearly separated into two clusters. A higher level of group separation and a better understanding of classification variables were applied by Supervised OPLS-DA. Table 1 and OPLS-DA scores plots. Figure 1C,D show the characteristics of the generated models. Groups J and Y achieved good separation of lipid extracts. A loading plot was constructed based on the OPLS-DA, which showed the contribution of variables to the differences observed among samples (Additional file 1: Fig. S2).

**Table 1:** OPLS-DA Model Summary for the discrimination between groups J and Y, using cross-validation.

Model	Type	A	N	R2X (cum)	R2Y (cum)	Q^2^ (cum)	Title
M5	OPLS-DA	1 + 1 + 0	12	0.701	0.986	0.958	J VS Y (POS)
M5	OPLS-DA	1 + 1 + 0	12	0.805	0.992	0.977	J VS Y (NEG)

The R2X and R2Y values show the total variation in the X and Y matrix explained by the models, respectively. The Q^2^ value represents the model predictability and statistical validity.

### TAGs Diversity and Fatty Acyl Chains Associated with TAG Under Pupal State

**Figure 2:**
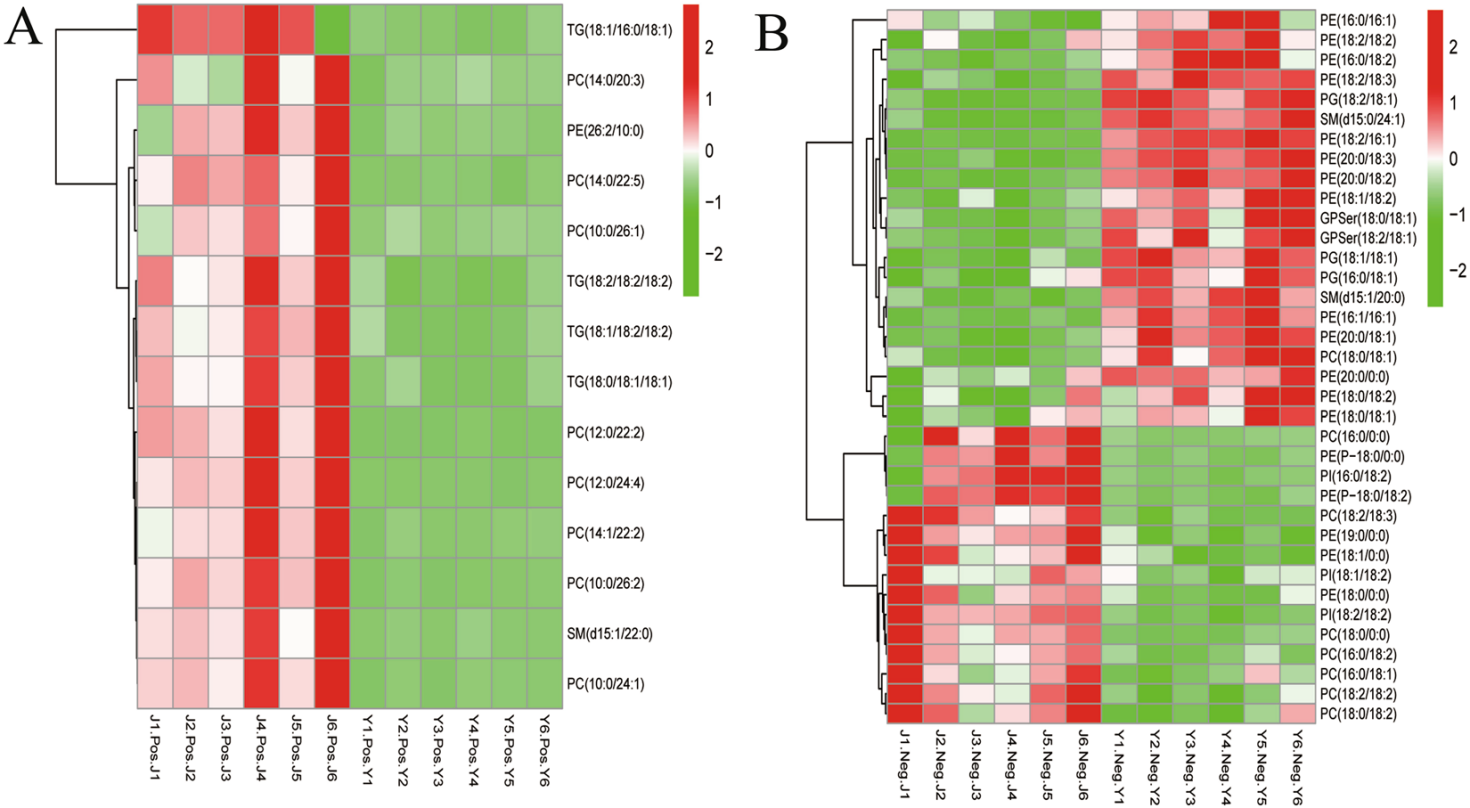
Integration of intermatrix lipid correlations between J and Y. Significantly changed metabolites in the lipid species of groups J and Y from GC-MS were analyzed by heat map. Heat maps were applied to display the relative quantity of each lipid species, in which a gradient of red colors represents higher quantity and a gradient of blue colors represents lower quantity in J compared with Y. (**A**) Heat map of J vs Y (POS). (**B**) Heat map of J vs Y (NEG). (VIP > 1, p < 0.05).

**Figure 3:**
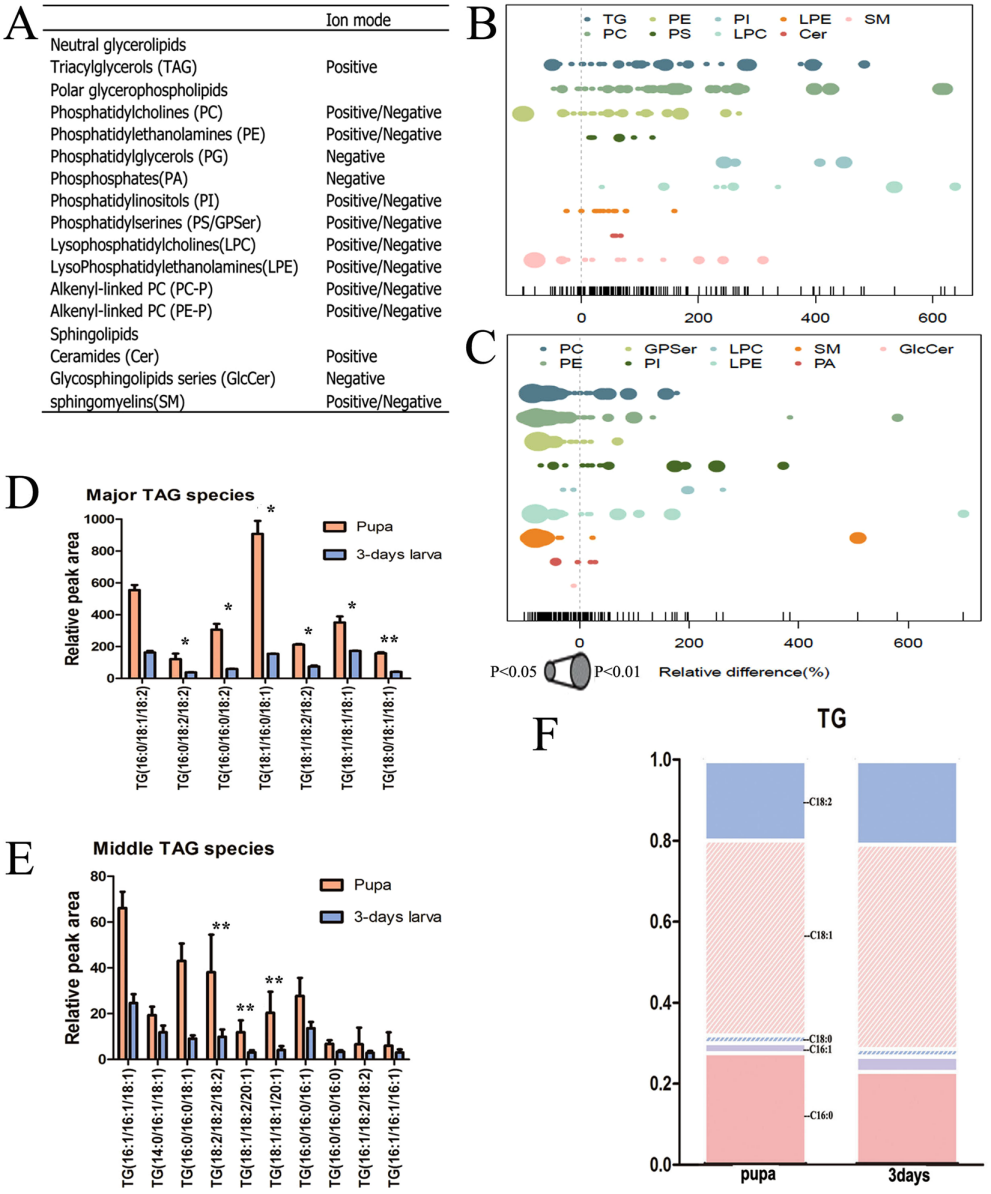
Changes in lipid composition and TAG fatty acyl chains during *L*. *japonica* pupation (middle or minor were determination by abundance). (**A**) Lipid classes identified in lipidomics experiments and their abbreviations as used in this article. (**B**,**C**) The relative percentage differences among all quantified lipid species between pupae and 3-days-old larvae (POS and NEG). Each dot represents a lipid species, and dot size indicates significance. Different colors represent different lipid classes. n = 6 for both groups. (**D**,**E**) The relative peak area of quantified lipid classes in major TAG (**D**) and middle TAG (**E**) in pupae and 3-days-old larvae. Data is presented as means + SEM; n = 6 for both groups. Significance level: fold change >1.5 or <0.5, and p < 0.05, *p < 0.05, **p < 0.01. The relative peak area of quantified lipid classes in minor TAG is shown in Fig. S3. (**F**) Glycerolipid base chemistry and fatty acyl chains changes among major TAG in pupae and 3-days-old larvae. Data is presented as means + SEM; n = 6 for both groups.

Significant metabolic changes among lipid species are represented as heat maps in Fig. 2 (VIP > 1, p < 0.05). In most analyzed lipid classes, relative differences revealed significant changes in the abundance of specific lipid species (Fig. 3). As shown in Fig. 2, the lipid composition is dynamic between the two tested stages. In the pupal stage (group J), TAGs increased significantly compared with the larval stage (group Y). More detailed classification for all lipid classes presented in Fig. 3A reveals significant changes in the abundance of most lipid classes analyzed in this study.

**Figure 4:**
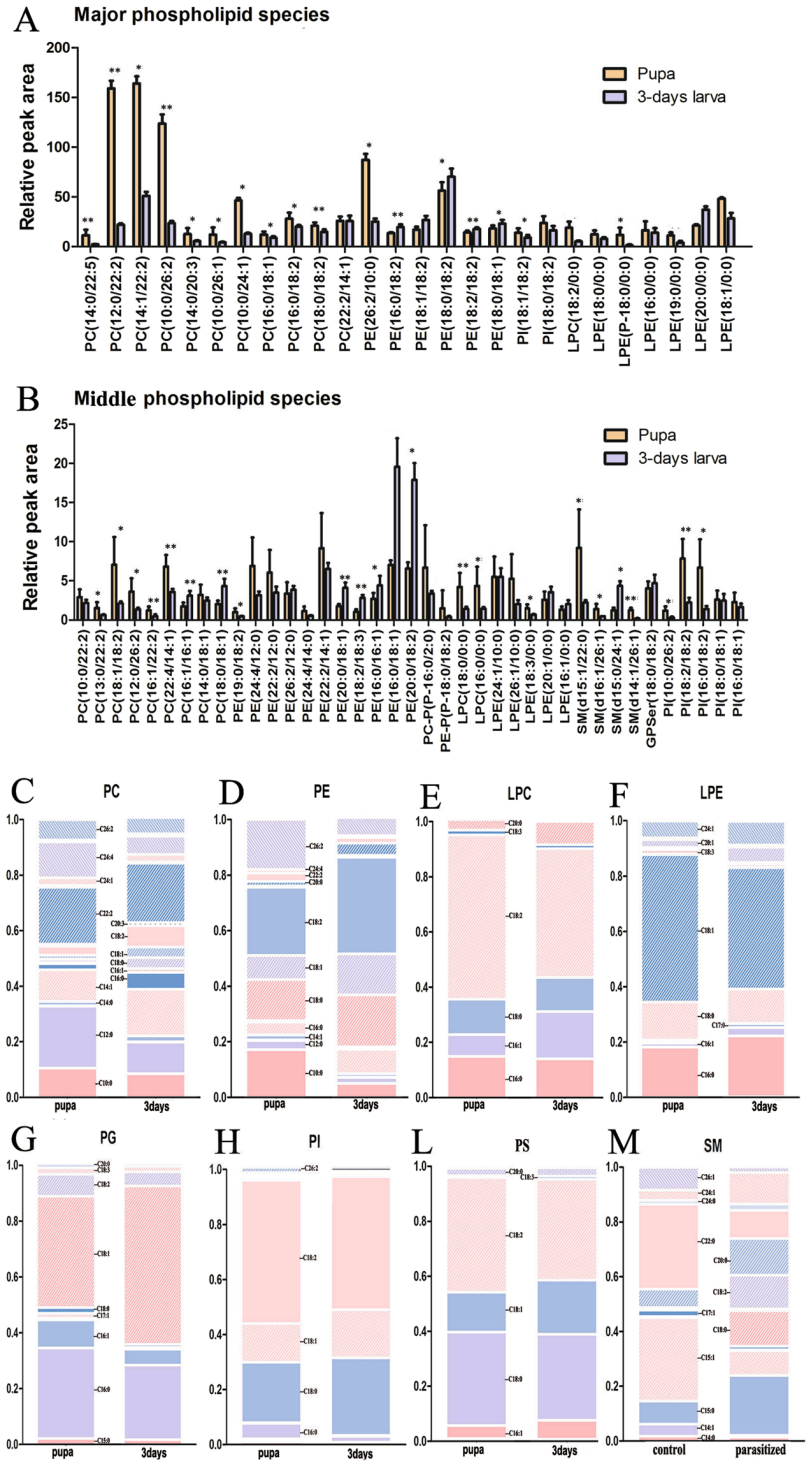
Changes in lipid composition and fatty acyl chains of PL during *L. japonica* pupation. (**A**,**B**) The relative peak area of quantified lipid classes in major PL (**A**) and middle PL (**B**) in pupae and 3-days-old larvae. Data is presented as means + SEM; n = 6 for both groups. Significance level: fold change >1.5 or <0.5, and p < 0.05, *p < 0.05, **p < 0.01. The relative peak area of quantified lipid classes in additional middle and minor PLs is shown in Fig. S3. (**C**–**M**) Glycerophospholipid base chemistry and fatty acyl chains changes of PL in pupae and 3-days-old larvae (PC, C;PE, D;LPC, E;LPE, F;PG, G;PI, H;PS, L; SM, M). Data is presented as means + SEM; n = 6 for both groups.

However, there were numerous changes in species composition (Fig. 3; Additional file 2) and in the total concentration of individual fatty acyl chains associated with TAG (Fig. 4F). All TAGs (e.g., (TAG (16:0/18:1/18:2), TAG (16:0/16:0/18:2), TAG (18:1/16:0/18:1)) (Fig. 3D,E) in the pupal stage increased markedly relative to the larval stage. For example, TAG (18:1/16:0/18:1) increased 5.8-fold, and TAG (18:2/18:2/18:2) increased 3.8-fold. Conversely, no increases or decreases were observed in the levels of fatty acyl chains associated with TAGs. Furthermore, C16:0, C16:1, C18:1, and C18:2 (Fig. 3F) of major lipid classes were only marginally affected in the pupal stage compared to the larval stage.

### Glycerophospholipids Up-regulated in *L. japonica* Pupal stage

Changes of the composition on different glycerolphospholipid classes were analyzed in the pupal stage. Significant changes in PLs are shown in a heat map (Fig. 2 (VIP > 1, p < 0.05)). Although pupation did not alter the abundance of the different glycerophospholipid classes, the numbers of major and middle glycerophospholipid species decreased (Fig. 4, Additional file 1: Fig. S3).

Lipidomics also revealed complex specificity in lipid metabolism. Compared to the larval stage, the vast majority of phospholipids significantly increased in pupae (e.g., PC (phosphatidylcholines (14:0/22:5)), 4.9-fold; PI (phosphatidylinositols (18:2/18:2)), 3.5-fold; SM (sphingomyelins (d15:1/22:0)), 4.0-fold; LPC (lysophosphatidylcholines (18:0/0:0)), 2.9-fold; PE-P (alkenyl-linked PE (P-18:0/0:0)), 8.0-fold). However, almost all phosphatidylethanolamines (PEs) (e.g., PE (18:2/16:1), 0.1-fold) and the majority of phosphatidylserines (PSs) (e.g., GPSer (18:0/18:1), 0.3-fold) significantly decreased in the pupal stage relative to the larval stage (Fig. 4A). Additionally, when analyzing fatty acyl chains associated with glycerophospholipids, a marked reduction in C18:2 and only marginal increases in C16:0 and C16:1 (Fig. 4) were detected. Increases in C16:0 and C16:1 corresponded with higher LPC and LPE (lysophosphatidylethanolamines) (Fig. 4E,F). However, C18:2 levels decreased with elevated levels of LPE, LPC, PI and PG (Fig. 4A,E,F,G,H), and with decreased levels of PS and PE (Fig. 4B,L). In these analyses, the direct effects of fatty acyl chains remolding during pupation could not be excluded. When quantified for individual glycerophospholipid subclasses, a marked reduction in C18:1 and increase in C18:0 in species PE and PS (Fig. 4B,L) were detected and significant increases in C18:1 in species PI and PG (phosphatidylglycerols) were observed. Based on these results we concluded that the changes in acyl chain composition in glycerophospholipids were associated with distinct *L*. *japonica* subclasses.

Thus, these changes confirmed that not only the content of glycerophospholipid subclasses changed, but glycerophospholipid fatty acyl chains remolded for new tissue and cell membrane construction.

### Gene Expression Related to Lipid Metabolism

**Figure 5:**
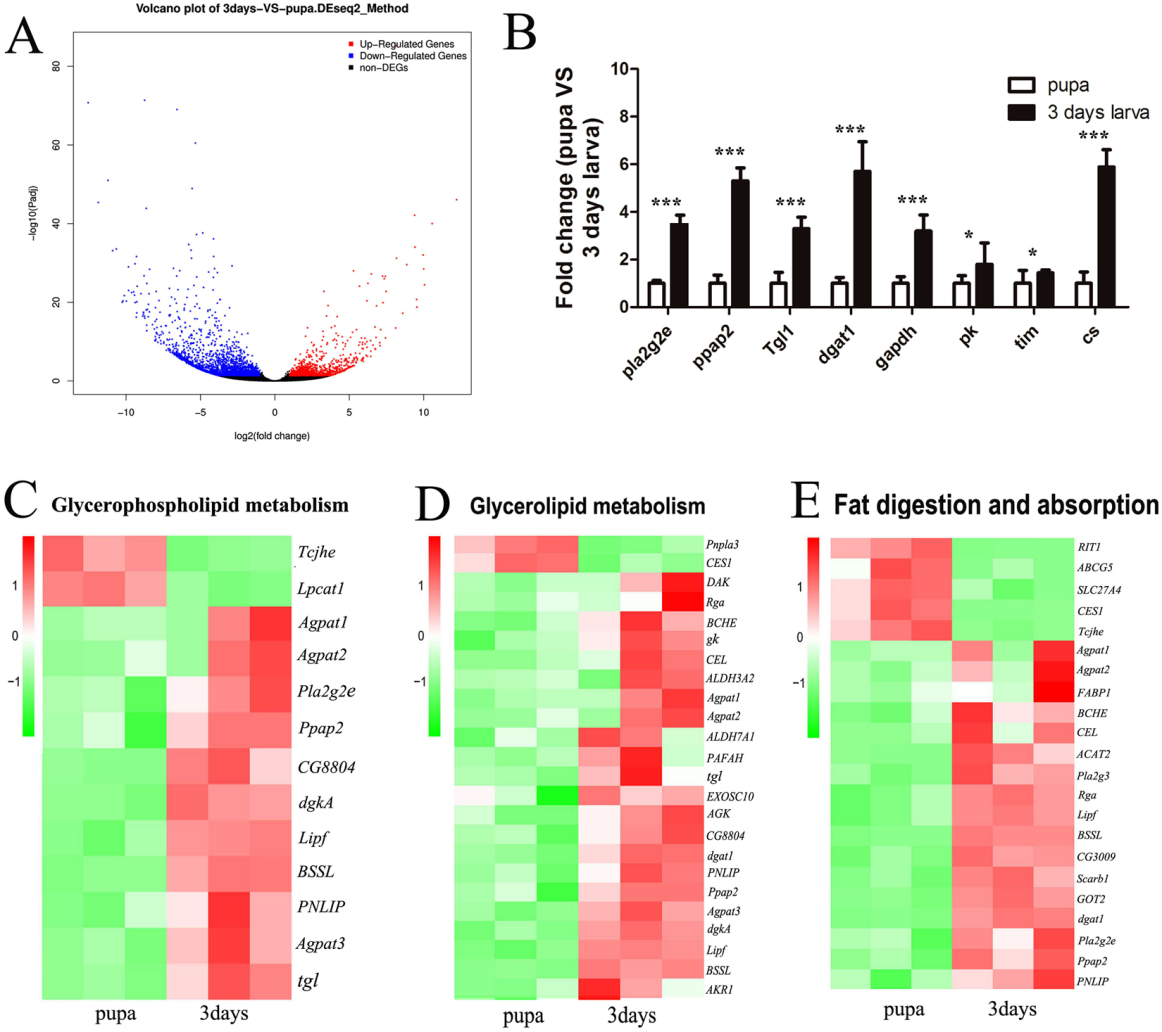
Pupation of *L*. *japonica* induces expression of genes involved in lipid metabolism. Total RNA obtained from both larvae and pupae was purified for RNA-seq. Differential mRNA expression between samples was determined with Fold change and *p*-value. (**A**) Log2 fold changes in RefSeq gene bodies in larvae and pupae and the corresponding significance values displayed as fold change >2 or <0.5 and *p* value < 0.05. The x-axis represents −log10 transformed significance, and the y-axis represents log2 transformed fold change. Red points represent up-regulated DEG. Blue points represent down-regulated DEG. Black points represent non-DEGs. *Pk*, pyruvate kinase; *cs*, citrate synthase; *tgl*, triacylglycerol lipase; *gapdh*, glyceraldehyde-3-phosphate dehydrogenase; *tim*, triosephosphate isomerase. (**B**) Relative expression levels of genes from the RNA-seq data of J and Y. Data is a representative set (three) containing duplicate samples. Significance levels: ***Fold change >2 or <0.5 and *p* value < 0.001. (**D**,**E**) Heat map representation of gene expression changes for significantly enriched metabolic KEGG pathways during pupation (glycerophospholipid metabolism, (**C**) glycerolipid metabolism, (**D**) fat digestion and absorption (**E**). The log10 expression values for each sample were clustered and uniformed in rows. *Tcjhe*, juvenile hormone esterase; *LPCAT1*, lysophosphatidylcholine acyltransferase; *CG8804*, similar to CG8804-PA; dgkA, diacylglycerol kinase; *Lipf*, lipase; *BSSL*, bile salt-stimulated lipase; *PNLIP*, pancreatic triacylglycerol lipase; *Pnpla3*,patatin-like phospholipase domain containing 3;*CES1*, carboxylesterase 1;*DAK*, bifunctional ATP-dependent dihydroxyacetone kinase;*Rga*, regulator of gene activity protein;*BCHE*, cholinesterase;*CEL*, esterase FE4-like; *ALDH3A2*, aldehyde dehydrogenase family 3 member A2; *ALDH7A1*,aldehyde dehydrogenase family 7 member A1; *EXOSC10*,exosome component 10; *AGK*,acylglycerol kinase; *AKR1*,aldo-keto reductase 1; *RIT1*, tRNA A64-2′-O-ribosylphosphate transferase; *ABCG5*,ATP-binding cassette sub-family G member 5; *SLC27A4*,solute carrier family 27; *FABP1*,fatty acid-binding protein 1; *ACAT2*,acetyl-CoA C-acetyltransferase; Pla2g3,phospholipase A2, group III;*CG3009*, CG3009 gene product from transcript CG3009-RA; *Scarb1*, scavenger receptor class B member 1; *GOT2*, aspartate aminotransferase.

Using a significance level of *p* < 0.05, a total of 1,159 differentially expressed genes, of which 463 show up-regulated (>2-fold) and 696 down-regulated (<0.5-fold) expression (Fig. 5A) were detected. All differentially expressed genes are shown in Additional file 3. This suggests an effect of pupation on lipid gene expression.

Analyses of the differentially expressed genes (DEG) in 3-days-old larvae using the Kyoto Encyclopedia of Genes and Genomes (KEGG) pathways^13,17^ revealed significant enrichment in lipid metabolic pathways and key nutrient metabolic pathways involved in lipid synthesis. These include glycerophospholipid metabolism, glycerolipid metabolism and fat digestion and absorption (Fig. 5C,D,E), the Krebs cycle (Additional file 1: Fig. S1A) and glycolysis (Additional file 1, Fig. S1B). Genes related to these pathways include phospholipase A2 (*pla2g2e*), phosphatidate phosphatase (*ppap2*), and diacylglycerol O-acyltransferase 1 (*dgat1*) (Fig. 5B). The most highly enriched pathway for down-regulated genes was the MAPK pathway (Additional file 1, Fig. S1C). Moreover, we found extensive changes in the 3-days-old larval transcriptome compared to the pupal transcriptome. These changes included up-regulation in the expression of genes involved in lipid metabolism.

### Gene Transcription in Key Nutrient Metabolic Pathways Involved in Lipid Synthesis

**Figure 6:**
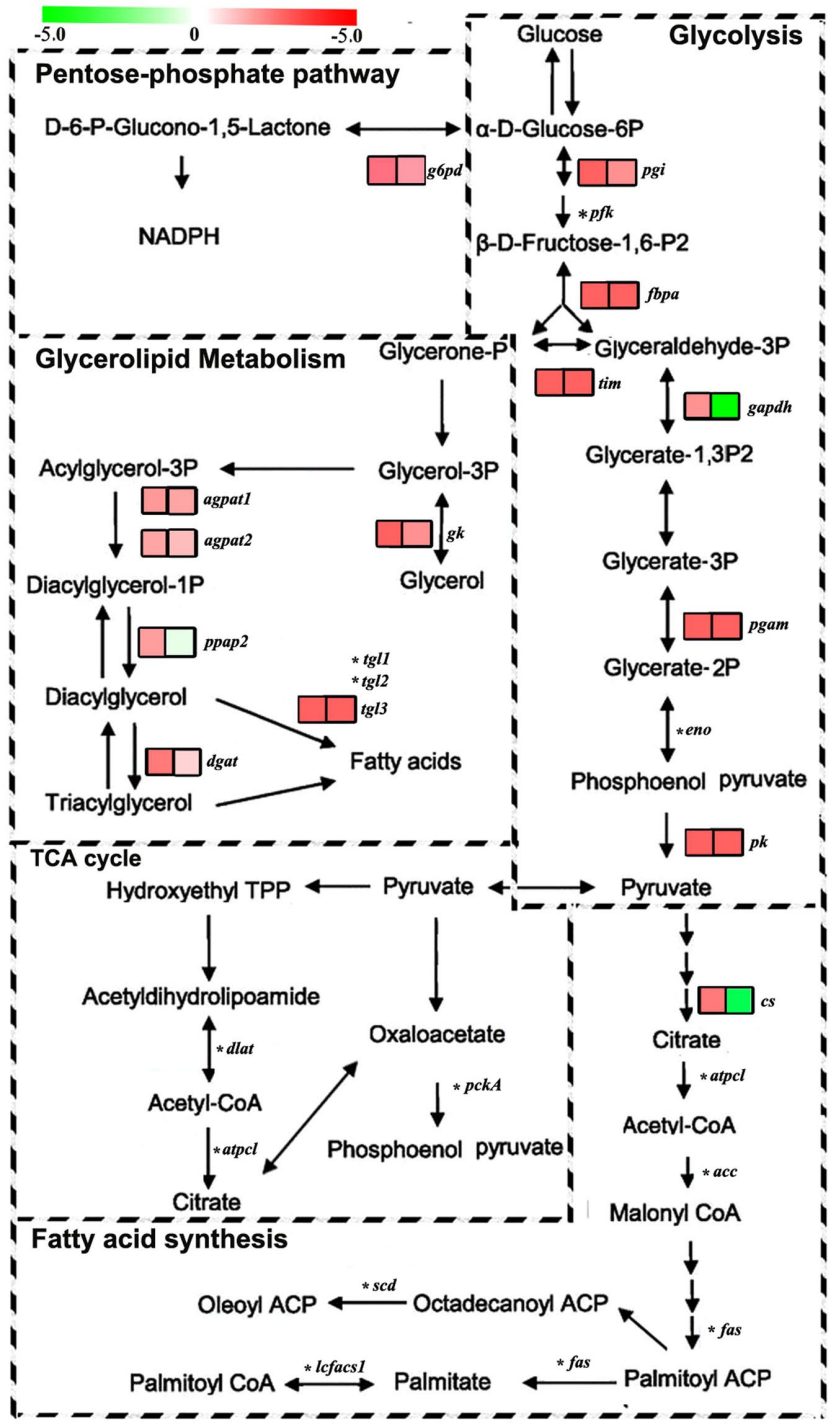
Gene transcription in key nutrient metabolic pathways involved in lipid synthesis between *L*. *japonica* pupae and 3-days-old larvae. Selected genes involved in KEGG lipid synthesis with indications of quantified lipid classes and genes significantly regulated during pupation. Red and green colors indicate up- and down-regulated gene expression, respectively. Log10 of the gene expression in RefSeq are shown in larvae (left) and pupae (right). Genes in the glycolytic pathway and TCA cycle were also selected, as they stimulate TAG synthesis pathways in larvae. *fbpa*, fructose 1,6-bisphosphate aldolase; *dlat*, dihydrolipoamide acetyltransferase component of pyruvate dehydrogenase; *pgam*, phosphoglycerate mutase. *The genes which FPKM were below 1, it was too low for comparison.

At the transcription level genes involved in the nutrient metabolic pathways in *L*. *japonica* were significantly different following pupation compared to 3-days-old larvae (Fig. 6, Additional file 1). Pupation reduced the transcription levels of phosphoglucose isomerase (*pgi*) (Fig. 6) involved in carbohydrate metabolism, and located at the beginning of the glycolytic pathway. Enolase (*eno*), a metalloenzyme responsible for catalyzing the conversion of 2-phosphoglycerate (2-PG) to phosphoenolpyruvate (PEP), showed a decrease in transcript levels (Fig. 6). Further support is provided by the lower abundance of glucose-6P dehydrogenase (*g6pd*) of the pentose phosphate pathway in pupae, compared with 3-days-old larvae of *L*. *japonica*. Furthermore, the rate-limiting enzyme, *pk*, was expressed less in pupae than in 3-days-old larvae, indicating a decrease in acetyl-CoA levels from sucrose. Conversely, 5 genes significantly decreased in pupae compared to 3-days-old larvae: glycerol kinase (*gk*), 1-acyl-sn-glycerol-3P acyltransferase 1/2 (*agpat1/2*), *ppap2*, and *dgat* (Fig. 6). Among these, *agpat* catalyzes the conversion of acylglycerol-3P to diacylglycerol-1P for use as glycerolipid components of cell membranes, while *dgat* catalyzes the synthesis of triglycerides from diacylglycerol. In TCA cycle, *cs* showed decreased transcriptional activity in *L*. *japonica* pupae (Fig. 6). Citrate synthase performs the first step in the TCA cycle, and down-regulation of the TCA cycle is consistent with reduced energy metabolism during pupation. The expression of several genes involved in TCA cycle were too low for comparison, including *pckA* (phosphoenolpyruvate carboxykinase) and ATP citrate lyase (*atpcl*), the FPKM values of which were below 1. Additionally, LSD (lipid storage droplet), which is specifically involved in the activation of triglyceride lipolysis in fat body adipocytes^18^, were up-regulated in 3-days-old larvae relative to pupae. Finally, neither 3-days-old larvae nor pupae expressed any of the key genes involved in fatty acid synthesis in abundance (eg., *acc* (acetyl-CoA carboxylase), *fas* (fatty acid synthase), *scd* (stearoyl-CoA desaturase), and *lcfacs1* (long-chain fatty acyl-CoA synthetase)) (Fig. 6). Based on these data, we suggest that *L*. *japonica* does not possess the ability for *de novo* fatty acids synthesize.

## Discussion

Research suggests that parasitoid wasps do not synthesize lipids^7–10,19–22^. Studies have also shown that mortality to wasps in highly resistant aphids typically occurs <72 h after oviposition, the host aphid is killed on day 6 or 7, and the adult parasitoids emerge 12 days after oviposition^23–25^. We observed that *L*. *japonica* had shorter developmental periods than the other parasitoids; 72 h after oviposition, *L*. *japonica* was only in the 2–3 instar larval stage. Although studies conducted on parasitoids have primarily focused on life-history and the physiological and biochemical changes in hosts^15,26,27^, little is known about the changes in lipidomics and transcriptional regulation in *L*. *japonica*. Lipids change in *L*. *japonica* is a multistep process that entails a complex interplay between metabolic and transcriptional signaling pathways. In this study, mass spectrometry-based lipidomics and RNA-seq were applied to provide a resource to study these changes at the level of the lipidome and transcriptome.

Our data demonstrated that TAGs and glycerophospholipids are not only modulated differentially, but phospholipid subspecies are also selectively remodeled during pupation. Due to their roles in various metabolic processes and being integral to maintaining metabolic homeostasis, differences in lipid content and fatty acyl chain composition during *L*. *japonica* development are valuable resources. Previous studies reported that the total amount of lipids increased during the pupal stage relative to the larval stage^2,28–30^. This study showed that *L*. *japonica* has a significant effect on *A*. *gossypii* lipid synthesis^15^. The abundance of TAGs in mummified aphids was significantly higher than in 3-days-old larvae. During the pupal stage metabolism was slow with almost no life activities, increased material metabolism, and signal transduction was not required^2^. The higher amount of TAGs in pupae suggested that TAGs were not utilized as a source of energy via beta oxidation. Instead, deposited TAGs may be required for tissue remodeling during pupal development^31^. Furthermore, this increase likely enhanced changes in membrane composition^32^. Our data also demonstrated that almost all phospholipids increased during the pupal stage, except for PE and the majority of PS (Figs 3B,C and 4A,B). Changes in lipid stores occurred because of flight, mating activities, and oviposition^30,33–35^. These changes closely correlate with the physiological needs of insects and differ considerably between species, sex, age, and developmental stage, and are influenced by environmental factors^36–39^. However, it is possible that these changes also reflect the differential effect of *L*. *japonica* developmental stages on the affinities of enzymes involved in remodeling and glycerophospholipid and TAG synthesis^40^.

We also observed significant glycerophospholipid remodeling in pupae compared to larvae. Similar findings were reported for RAT^41^. In the analysis of fatty acyl chains associated with TAG, we detected only the effect on TAG species. However, when analyzing the total pool of fatty acyl chains associated with glycerophospholipids, a marked reduction in C18:2 (Fig. 4) were detected. Furthermore, differential retention of acyl chains in different phospholipid subclasses and subspecies occurred during different developmental stages. Therefore, one key finding of this study is that pupation adaptation entails the remodeling of TAG and glycerophospholipids in a highly subspecies-selective manner. Future studies are required to determine the functional consequences of this remodeling in *L*. *japonica*.

Based on significant lipidome changes between stages, we focused our transcriptome analyses on key lipid synthesis metabolic pathways. In *Rhodnius prolixus*, the glycerol-3-phosphate pathway is the only route for triacylglycerol synthesis^26^. *Pgi, fbpa*, and *gapdh* are involved in glycolysis, which consumes GAP to produce pyruvate, and were significantly up-regulated in 3-days-old *L*. *japonica* larvae (Fig. 6). This result synchronizes with parasitized 3-days-old aphids^15^. Additionally, *g6pd*, a TCA cycle transcript, producing the reducing agent nicotinamide adenine dinucleotide phosphate (NADP) in the pentose phosphate pathway, was down-regulated in pupae (Fig. 6). Transcription levels of these key nutrient metabolic pathways genes were in line with the high abundance of TAG in pupae. In conclusion, we have demonstrated that relative to pupae, 3-days-old *L*. *japonica* larvae have up-regulated expression of key nutrient metabolic pathway genes involved in lipid synthesis. Furthermore, genes involved in the MAPK signal pathway, which is essential for lipid homeostasis^42^, were up-regulated in pupae (Fig. S1C).

Our data showed a robust accumulation of numerous TAGs in pupae. This conversion did not change the lipid class, but changed lipid content and the fatty acyl chains associated with TAG species (Fig. 3), and increased expression of genes encoding enzymes involved in TAGs synthesis. Some enzymes that catalyze reversible reactions in the glycerolipid synthesis pathway, such as *gpat* (glycerol-3-phosphate O-acyltransferase) and *dgat*, can be regulated at the transcriptional level in mammals^43^. Among the analyzed genes involved in the glycerolipid pathway *gk*, *ppap2*, *agapt*, and *dgat* were significantly down-regulated in pupae relative to 3-days-old larvae (Fig. 6). We presume that TAG synthesis and accumulation primarily occurred in larvae and then broke-down in the pupal stage. Studies have shown that insect lipids increase because of absorption or synthesis from food material during larval stages^33,44–46^. Many insects were also shown to deposit large lipids, and metabolize them during pupal-adult transformation for new synthesis, cellular changes, and tissue remolding^2,29^. Further support for this was provided by the higher transcription of genes involved in fat digestion and absorption in 3-days-old *L*. *japonica* larvae, relative to pupae. Furthermore, higher transcript abundance of *lsd*, a gene specifically involved in the activation of triglyceride lipolysis in fat body adipocytes^18^, was found in pupae than in 3-days-old larvae. With the increase in triglyceride synthesis in 3-days-old larvae, *lsd* was significantly up-regulated in pupae.

In this study, the transcript abundance of lipid synthesis genes indicates that fatty acid synthesis may not occur in *L*. *japonica*, at least not in the larval or pupal stages. Detectable transcript levels were observed for *atpcl*, *dlat*, *acc*, *fas*, *scd*, and *lcfacs1* in either larvae or pupae (Fig. 6). This result is in contrast to the high expression of these genes in parasitized aphids^15^. *Acc* and *fas* are 2 key genes for which active gene transcription is crucial for fatty acid synthesis^47–49^. The most important function of *Acc* is to provide the malonyl-CoA substrate for fatty acid biosynthesis^50^, while *fas* encodes the enzyme that performs the majority of steps involved in fatty acids synthesis^51^.

Many factors could have resulted in parasitoids losing the ability to synthesize fatty acids. For example, mutations accumulating in the *fas* promoter or genes encoding transcription factors could impair the lipogenic ability of parasitoids^52^. Alternatively, this may be an evolutionary choice stemming from lifestyle^53^. Our findings provide strong evidence that *L*. *japonica* neither synthesizes fatty acids nor balances increased catabolism of fatty acids. However, we cannot conclude that *L*. *japonica* is unable to perform lipid synthesis, as more studies are needed to investigate the molecular mechanisms underlying the lack of fatty acids synthesis, and to determine whether *L*. *japonica* has the capability to synthesize lipid and fatty acids in adult life.

## Conclusions

In summary, the changes in the lipidome and transcriptome of 3-days-old *L*. *japonica* larvae and pupae using advanced omics techniques were first determined. The results showed large amounts of TAGs being absorbed and synthesized in 3-days-old larvae, resulting in the accumulation of TAGs and PLs in pupae. This was accompanied by highly subspecies-selective TAG and glycerophospholipids remodeling, and a marked increase in the expression of genes involved in TAG synthesis and key nutrient metabolic pathways involved in lipid synthesis. Results also showed that neither larvae nor pupae possess the ability for fatty acid synthesis. Together, our data showed that lipidome and transcriptome changes between *L*. *japonica* pupae and 3-days-old larvae constitute a comprehensive and valuable resource for further studies on metabolic regulation and the molecular mechanisms underlying parasitic responses.

## Methods

### Insects

Adult L. japonica were collected from the Institute of Cotton Research of Chinese Academy of Agricultural Sciences, and reared in the laboratory on cotton-melon aphids at 24 ± 1 °C, a 14:10 h light/dark (L:D) photoperiod, and 75 ± 5% relative humidity (RH). Third instar cotton-melon aphids from the same mother were exposed to wasps, and the parasitized aphids were collected to separate wasp larvae after 3 days. Other parasitized aphids were reared on cotton leaves and collected 5 days later, when they became mummified alates. After dissection, the 3-days-old larvae (named group Y) and mummified (named group J) *L*. *japonica* were cleaned with ultrapure water and stored at −80 °C.

### Lipid extraction, identified and quantified

The 3-days-old and mummified *L. japonica* were prepared to extract lipid metabolites, each group had 6 biological repetitions with a total of 12 samples. 20 mg of each *L*. *japonica* sample was used. Lipids were extracted as described in our previous study^54^. During the LC/MS experiment, to acquire MS/MS spectra on an information-dependent basis (IDA), TripleTOF mass spectrometer was used. In this mode, the acquisition software (Analyst TF 1.7, AB Sciex) continuously evaluates and triggers the acquisition of MS/MS spectra. ProteoWizard was used to convert the raw data into mzXML and further analysis was performed using XCMS. For metabolite identification, accurate mass search (<30 ppm) and an in-house standard MS/MS library was used for MS/MS spectral match. Using the interquartile range denoising method, peaks were detected and metabolites were isolated. Half of the minimum detectable value was filled with the missing values. Molecular lipids were analyzed in both positive and negative ion modes using multiple precursor ion scanning-based methods^55,56^. Molecular lipid species were identified and relatively quantified (TAG, PC, PE, PS, LPC, LPE, PG, SM, PI, PA, PC-P, and PE-P)^57^. Lipids are referred to according to LIPID MAPS lipid nomenclature recommendations.

### Lipidomics Statistical Analysis

We used the area normalization method, with the total area of each sample as the denominator, the sample of the test substance divided by this total area. To account for group separation and classification variables, principal component analysis (PCA) and orthogonal projections to latent structures-discriminate analysis (OPLS-DA) were performed using the SIMCA14 software package (Umetrics, Umea, Sweden). A loading plot was constructed based on OPLS-DA, which showed the contribution of variables to differences between the groups. Heat maps were used to intuitively illustrate differences among these components. To refine this analysis, the first principal component of variable importance projection (VIP) was obtained. Changed metabolites were determined by the Student’s *t* test (p > 0.05) and VIP values exceeding 1.0^58,59^. Additionally, another criterion for compounding differences (fold change > 1.5 or <0.5, and p < 0.05) was used. The relative difference (percent) of samples was conducted by Hodges-Lehmann estimator. A complete list of the lipidomics data is provided in Additional file 2. The Metabolights number is MTBLS406.

### RNA Extraction

Transcriptome analysis was performed to investigate the changes in lipid composition at the level of gene expression. Since *L*. *japonica*’s pupation period is about 5 days, consistent with lipidomics, we chose an intermediate transition period of 3 days and the early stage of pupation to verify gene transcriptional changes. Each group had 3 biological repetitions. Total RNA samples were extracted from 3-days-old wasp larvae and mummified *L*. *japonica* using TRIZOL reagent (Invitrogen, Carlsbad, CA, USA) following the manufacturer’s instructions. Illumina sequencing was performed using the Illumina HiSeq. 2500 system at the Beijing Genomics Institute in Shenzhen, China.

### RNA-Seq Data Analysis

The sequencing reads were first processed to remove low-quality, adaptor-linked, and high content of unknown base (N) reads, prior to downstream analyses. Transcriptome *de novo* assembly was carried out with the short read assembling program Trinity^60,61^, with min_kmer_cov set to 2 by default, and all other parameters also set to default. Resulting assembled sequences were considered unigenes. Unigenes larger than 150 bp were compared using BLASTX to protein databases including NCBI-nr, Swiss-Prot, KEGG, and COG (e-value < 10^−5^), Sequences were screened for preliminary identification and further functional annotation by BLASTX in NCBI-nr and Swiss-Prot and BLASTN in NCBI-nt (e-value < 10^−5^). Then, the Blast2GO program^62^ was used to obtain GO (Gene Ontology) annotation for the unigenes, and WEGO software^63^ to obtain GO functional classification. HOMER^64^ was used for functional enrichment analysis and KEGG database was used to annotate genes to metabolic pathway^13,17^. The reads per kilobase per million mapped reads (RPKM) method was used to estimate the abundance of each unigene, eliminating the influence of gene length and sequencing discrepancies in the calculations. Differential expression was determined using a cutoff significance level of *p* < 0.05 under a fold change >2 or <0.5. The sequencing data is submitted to the ArrayExpress database and has the accession number E-MTAB-5227.

### Availability of data and materials

The raw sequence datasets supporting the results of this article are available in the sequence read archive repository (http://www.ebi.ac.uk/arrayexpress/ and http://www.ebi.ac.uk/metabolights/) under the accession numbers E-MTAB-5227 and MTBLS406. Additional supporting tables are included as Additional files.

### Ethics approval and consent to participate

Samples in the study were collected from the Institute of Cotton Research of Chinese Academy of Agricultural Sciences. This article did not contain any studies with human participants or animals performed by any of the authors. No specific permits were required.

## Electronic supplementary material

Additional file 1

Dataset 1

Dataset 2

## References

[1] Stanley-Samuelson DW, Jurenka RA, Cripps C, Blomquist GJ, de Renobales M (1988). Fatty acids in insects: Composition, metabolism, and biological significance. Archives of Insect Biochemistry and Physiology.

[2] Beenakkers AMT, Bloemen REB, De Vlieger TA, Van Der Horst DJ, Van Marrewijk WJA (1985). Insect adipokinetic hormones. Peptides.

[3] Nawrot, J., Malinski, E. & Szafranek, J. In *Proceedings of the 6th international working conference on stored-product protection*. 553–560 (1996).

[4] Uner N (1988). Lipid composition of the fat body, haemolymph and muscle in Tenebrio molitor L.(Coleoptera: Tenebrionidae) larvae. Commun. Fac. Sci. Univ. Ank. Serie. C.

[5] Downer R, Matthews J (1976). Patterns of lipid distribution and utilisation in insects. American Zoologist.

[6] Leventis PA, Grinstein S (2010). The distribution and function of phosphatidylserine in cellular membranes. Annual review of biophysics.

[7] Olson D, Fadamiro H, Lundgren J, Heimpel GE (2000). Effects of sugar feeding on carbohydrate and lipid metabolism in a parasitoid wasp. Physiological Entomology.

[8] Fadamiro HY, Heimpel GE (2001). Effects of partial sugar deprivation on lifespan and carbohydrate mobilization in the parasitoid Macrocentrus grandii (Hymenoptera: Braconidae). Annals of the Entomological Society of America.

[9] Rivero A, West S (2002). The physiological costs of being small in a parasitic wasp. Evolutionary Ecology Research.

[10] Lee JC, Heimpel GE, Leibee GL (2004). Comparing floral nectar and aphid honeydew diets on the longevity and nutrient levels of a parasitoid wasp. Entomologia experimentalis et applicata.

[11] Visser B, Ellers J (2008). Lack of lipogenesis in parasitoids: A review of physiological mechanisms and evolutionary implications. Journal Of Insect Physiology.

[12] Visser B (2010). Loss of lipid synthesis as an evolutionary consequence of a parasitic lifestyle. Proceedings of the National Academy of Sciences of the United States of America.

[13] Kanehisa M, Goto S (2000). KEGG: kyoto encyclopedia of genes and genomes. Nucleic acids research.

[14] Xu J (2007). Dandruff-associated Malassezia genomes reveal convergent and divergent virulence traits shared with plant and human fungal pathogens. Proceedings of the National Academy of Sciences of the United States of America.

[15] Zhang S (2015). Effects of Lysiphlebia japonica (Ashmead) on cotton-melon aphid Aphis gossypii Glover lipid synthesis. Insect Mol Biol.

[16] Harkewicz R, Dennis EA (2011). Applications of mass spectrometry to lipids and membranes. Annual review of biochemistry.

[17] Kanehisa M (2014). Data, information, knowledge and principle: back to metabolism in KEGG. Nucleic acids research.

[18] Patel RT, Soulages JL, Hariharasundaram B, Arrese EL (2005). Activation of the lipid droplet controls the rate of lipolysis of triglycerides in the insect fat body. J Biol Chem.

[19] Ellers J, Van Alphen J (1997). Life history evolution in Asobara tabida: plasticity in allocation of fat reserves to survival and reproduction. Journal of Evolutionary Biology.

[20] Giron D, Casas J (2003). Lipogenesis in an adult parasitic wasp. Journal of Insect Physiology.

[21] Casas J (2003). Energy dynamics in a parasitoid foraging in the wild. J Anim Ecol.

[22] Visser B (2012). Transcriptional changes associated with lack of lipid synthesis in parasitoids. Genome biology and evolution.

[23] Martinez, A. J., Kim, K. L., Harmon, J. P. & Oliver, K. M. Specificity of Multi-Modal Aphid Defenses against Two Rival Parasitoids. *Plos One* **11**, 10.1371/journal.pone.0154670 (2016). 10.1371/journal.pone.0154670 PMC4852904 27135743

[24] Chow FJ, Sullivan DJ (1984). Developmental Stages of Praon pequodorum Viereck (Hymenoptera: Aphidiidae), a Pea Aphid Parasitoid. Annals of the Entomological Society of America.

[25] Danyk TP, Mackauer M (1996). An extraserosal envelope in eggs of praon pequodorum (hymenoptera, aphidiidae), a parasitoid of pea aphid. Biological Control.

[26] Alves-Bezerra M, Gondim KC (2012). Triacylglycerol biosynthesis occurs via the glycerol-3-phosphate pathway in the insect Rhodnius prolixus. Bba-Mol Cell Biol L.

[27] Sun Q (2011). Mammalian target of rapamycin up-regulation of pyruvate kinase isoenzyme type M2 is critical for aerobic glycolysis and tumor growth. Proceedings of the National Academy of Sciences of the United States of America.

[28] Pearincott JV (1960). Changes in the lipid content during growth and metamorphosis of the house fly, Musca domestica Linnaeus. Journal of cellular and comparative physiology.

[29] Agrell, I. P. & Lundquist, A. M. Physiological and biochemical changes during insect developm ent. *Physiol Insecta*, 159–247, 10.1016/B978-0-12-591601-1.50011-9 (1973).

[30] Nurullahoğlu ZÜ, Uçkan F, Sak O, Ergİn E (2004). Total lipid and fatty acid composition of Apanteles galleriae and its parasitized host. Annals of the Entomological Society of America.

[31] Guan XL (2013). Biochemical membrane lipidomics during Drosophila development. Developmental cell.

[32] Carvalho M (2012). Effects of diet and development on the Drosophila lipidome. Molecular systems biology.

[33] Gilby A (1965). Lipids and their metabolism in insects. Annual review of entomology.

[34] Fast PG (1964). Insect lipids: a review. Memoirs of the Entomological Society of Canada.

[35] Domroese LA, Gilbert LI (1964). The role of lipid in adult development and flight-muscle metabolism in Hyalophora cecropia. Journal of Experimental Biology.

[36] Guerra AA, Robacker DC (1989). Effects of sex, age, and diet on the triacylglycerol fatty acid composition of subtropical boll weevils, Anthonomus grandis Boheman (Coleoptera: Curculionidae). Journal of agricultural and food chemistry.

[37] Cohen WM, Levinthal DA (1990). Absorptive capacity: A new perspective on learning and innovation. Administrative science quarterly.

[38] Itoyama K, Tojo S, Yanagita T, Hardie J (2000). Lipid composition in long-day and short-day forms of the black bean aphid, Aphis fabae. Journal of Insect Physiology.

[39] BOZKUŞ K (2003). Phospholipid and triacylglycerol fatty acid compositions from various development stages of Melanogryllus desertus Pall.(Orthoptera: Gryllidae). Turkish Journal of Biology.

[40] Yamashita A (2014). Acyltransferases and transacylases that determine the fatty acid composition of glycerolipids and the metabolism of bioactive lipid mediators in mammalian cells and model organisms. Progress in lipid research.

[41] Marcher A-B (2015). RNA-Seq and Mass-Spectrometry-Based Lipidomics Reveal Extensive Changes of Glycerolipid Pathways in Brown Adipose Tissue in Response to Cold. Cell reports.

[42] Nunez LR (2008). Cell wall integrity MAPK pathway is essential for lipid homeostasis. J Biol Chem.

[43] Coleman RA, Lee DP (2004). Enzymes of triacylglycerol synthesis and their regulation. Progress in lipid research.

[44] Nestel D, Tolmasky D, Rabossi A, Quesada-Allué LA (2003). Lipid, carbohydrates and protein patterns during metamorphosis of the Mediterranean fruit fly, Ceratitis capitata (Diptera: Tephritidae). Annals of the Entomological Society of America.

[45] Fernando-Warnakulasuriya GJ, Tsuchida K, Wells MA (1988). Effect of dietary lipid content on lipid transport and storage during larval development of Manduca sexta. Insect Biochemistry.

[46] Arrese EL (2001). Lipid storage and mobilization in insects: current status and future directions. Insect biochemistry and molecular biology.

[47] Milgraum LZ, Witters LA, Pasternack GA, Kuhajda FP (1997). Enzymes of the fatty acid synthesis pathway are highly expressed in *in situ* breast carcinoma. Clinical Cancer Research.

[48] Kuhajda FP (2006). Fatty acid synthase and cancer: new application of an old pathway. Cancer research.

[49] Menendez JA, Lupu R (2007). Fatty acid synthase and the lipogenic phenotype in cancer pathogenesis. Nature Reviews Cancer.

[50] Abu-Elheiga L, Matzuk MM, Abo-Hashema KA, Wakil SJ (2001). Continuous fatty acid oxidation and reduced fat storage in mice lacking acetyl-CoA carboxylase 2. Science.

[51] Postic C, Girard J (2008). Contribution of de novo fatty acid synthesis to hepatic steatosis and insulin resistance: lessons from genetically engineered mice. The Journal of clinical investigation.

[52] Kunte AS, Matthews KA, Rawson RB (2006). Fatty acid auxotrophy in Drosophila larvae lacking SREBP. Cell metabolism.

[53] Mazumdar J, Striepen B (2007). Make it or take it: fatty acid metabolism of apicomplexan parasites. Eukaryotic cell.

[54] Gao, X. K. *et al*. Lipidomics and RNA-Seq Study of Lipid Regulation in Aphis gossypii parasitized by Lysiphlebia japonica. *Sci Rep* **7**, 10.1038/s41598-017-01546-1 (2017). 10.1038/s41598-017-01546-1 PMC5431011 28465512

[55] Ekroos K, Chernushevich IV, Simons K, Shevchenko A (2002). Quantitative profiling of phospholipids by multiple precursor ion scanning on a hybrid quadrupole time-of-flight mass spectrometer. Analytical chemistry.

[56] Ekroos K (2003). Charting molecular composition of phosphatidylcholines by fatty acid scanning and ion trap MS3 fragmentation. J Lipid Res.

[57] Ejsing CS (2006). Automated identification and quantification of glycerophospholipid molecular species by multiple precursor ion scanning. Analytical chemistry.

[58] Storey JD, Tibshirani R (2003). Statistical significance for genomewide studies. Proceedings of the National Academy of Sciences.

[59] Kind T (2009). FiehnLib: mass spectral and retention index libraries for metabolomics based on quadrupole and time-of-flight gas chromatography/mass spectrometry. Analytical chemistry.

[60] Grabherr MG (2011). Full-length transcriptome assembly from RNA-Seq data without a reference genome. Nature biotechnology.

[61] Li R (2010). De novo assembly of human genomes with massively parallel short read sequencing. Genome research.

[62] Conesa A (2005). Blast2GO: a universal tool for annotation, visualization and analysis in functional genomics research. Bioinformatics.

[63] Ye J (2006). WEGO: a web tool for plotting GO annotations. Nucleic Acids Res.

[64] Heinz S (2010). Simple combinations of lineage-determining transcription factors prime cis-regulatory elements required for macrophage and B cell identities. Molecular cell.

