# Supplementary material for: RNA-Seq and UHPLC-Q-TOF/MS Based Lipidomics Study in Lysiphlebia japonica
Source: Sci Rep. 2018 May 17;8:7802. doi: 10.1038/s41598-018-26139-4 (PMC5958133; doi:10.1038/s41598-018-26139-4)
Supplement: Supplementary file 1 — Additional file 1 [file 41598_2018_26139_MOESM1_ESM.docx]

**Supplementary Information**

RNA-Seq and UHPLC-Q-TOF/MS Based Lipidomics Study in *Lysiphlebia japonica*

**Gao Xueke, Luo Junyu, Lü Limin, Zhang LiJuan, Zhang Shuai*, Cui Jinjie** *****

***(Research Base , Anyang Institute of Technology, State Key Laboratory of Cotton Biology /Institute of***

***Cotton Research, Chinese Academy of Agricultural Sciences, Anyang, Henan 455000, China)***

This Supplementary Information contains:

[Additional file 1:](https://static-content.springer.com/esm/art%3A10.1186%2Fs13742-016-0136-3/MediaObjects/13742_2016_136_MOESM1_ESM.docx) Supplemental Experimental Procedures, Figures S1, S2 and S3, and Tables S3.

[Additional file 2:](https://static-content.springer.com/esm/art%3A10.1186%2Fs13742-016-0136-3/MediaObjects/13742_2016_136_MOESM2_ESM.docx) [Complete List of Lipidomics Data.](http://www.sciencedirect.com/science/MiamiMultiMediaURL/1-s2.0-S2211124715012589/1-s2.0-S2211124715012589-mmc3.xlsx/280959/html/S2211124715012589/6a92117806243bf25829aa83e29d0c33/mmc3.xlsx)

[Additional file 3: Different Changes in Gene expression between pupae and 3 days larvae in *L. japonica.*](http://www.sciencedirect.com/science/MiamiMultiMediaURL/1-s2.0-S2211124715012589/1-s2.0-S2211124715012589-mmc2.xlsx/280959/html/S2211124715012589/c4efd8d69c8add5b156ceaced7982ec4/mmc2.xlsx)


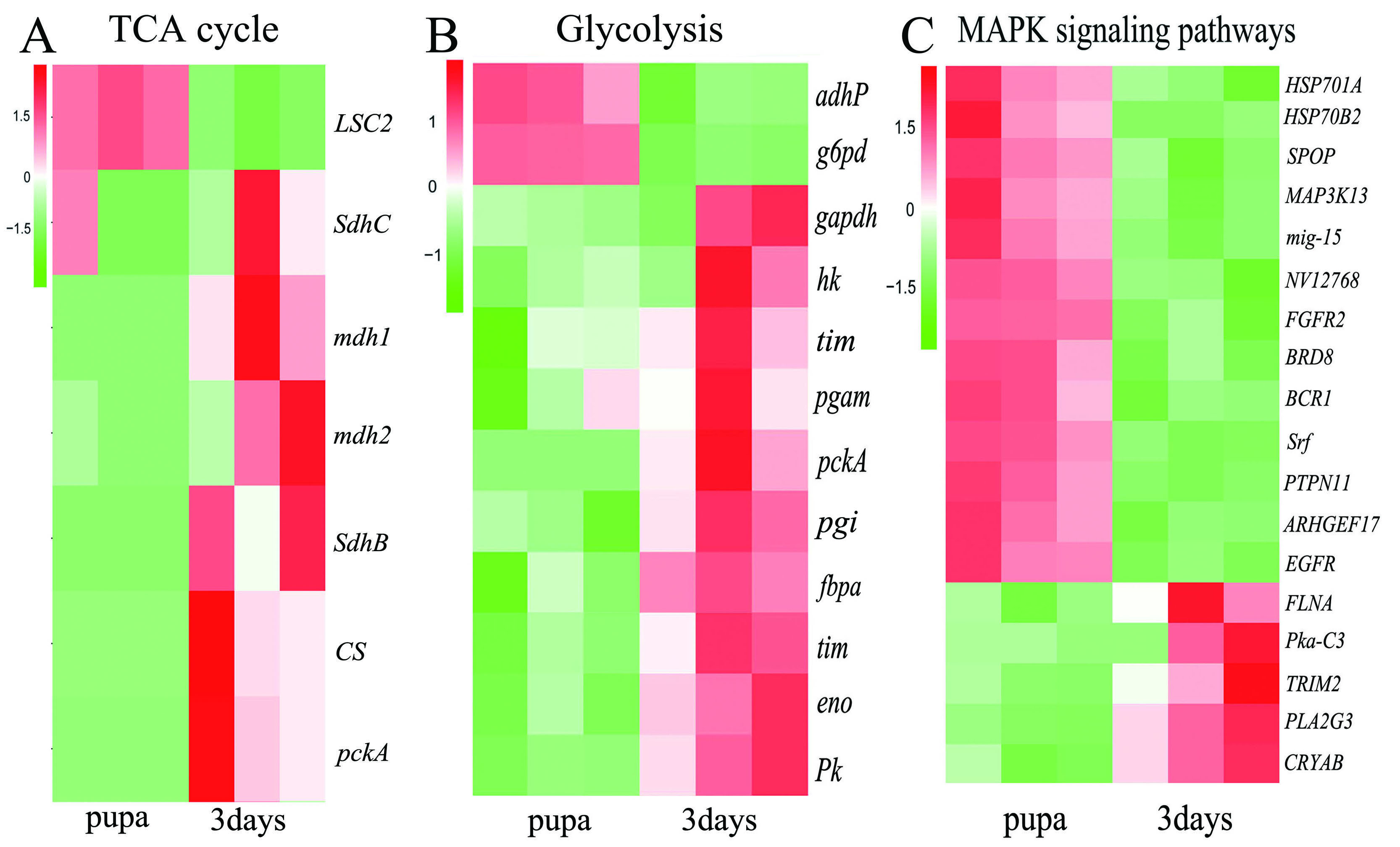


### Figure S1. Changes of Gene Expression Involved in Metabolism Pathway in 3 days larvae and pupae of [*L. japonica*](http://www.so.com/link?url=http%3A%2F%2Fwww.doc88.com%2Fp-9611679742241.html&q=%E6%A3%89%E8%9A%9C%E8%8C%A7%E8%9C%82&ts=1463193541&t=8cde705d94909f6d56063ab2923e2fa&src=haosou) *.*

### Heatmap represents the changes of genes expression that significantly enriched metabolic KEGG pathways between 3-days-old larvae and pupae (A, Citrate cycle (TCA cycle); B, Glycolysis; C, MAPK signaling pathway). The log10 expression values for each sample were clustered and uniformed in rows. *LSC2*, succinyl-CoA synthetase 2; *SdhC*, succinate dehydrogenase complex, subunit C; *mdh1*,malate dehydrogenase 1; *mdh2*, malate dehydrogenase 2; *SdhB*, succinate dehydrogenase iron-sulfur subunit; *adhP*, alcohol dehydrogenase, propanol-preferring; *hk*, hexokinasel; *HSP701A*, heat shock protein 70 A1; *HSP70B2*, heat shock protein 70 B2; *mig-15*, abnormal cell MIGration family member; *SPOP*, speckle-type POZ protein; *MAP3K13*, mitogen-activated protein kinase 13; *NV12768*, voltage-dependent calcium channel subunit alpha-2; *FGFR2*, fibroblast growth factor receptor 2; *BRD8*, bromodomain-containing protein 8; *BCR1*, breakpoint cluster region protein 1; *Srf*, serum response factor; *PTPN11*, protein tyrosine phosphatase, non-receptor type 11; *ARHGEF17*, Rho guanine nucleotide exchange factor (GEF) 17; *EGFR*, epidermal growth factor receptor; *FLNA*, filamin; *Pka-C3*,cAMP-dependent protein kinase 3; *TRIM2*, tripartite motif-containing protein 2; *CRYAB*, crystallin, alpha B.


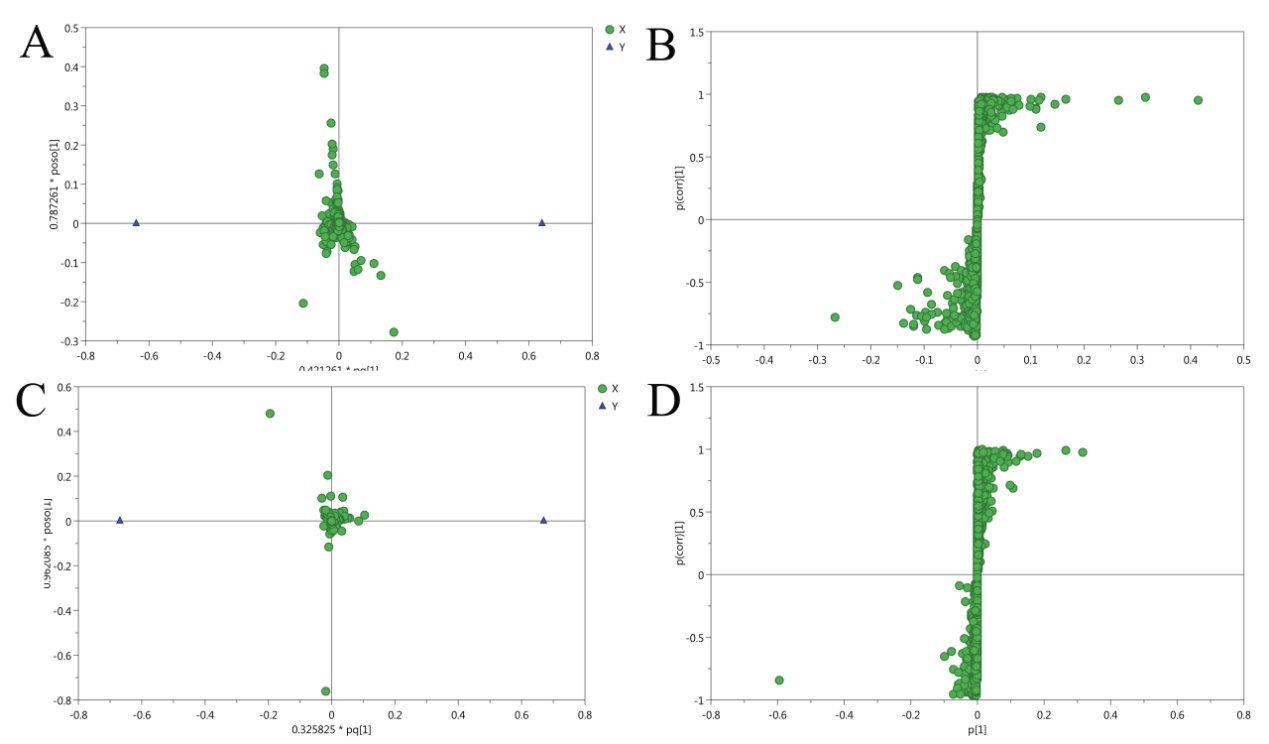


**Figure S2. Loading Scatter Plot and S-plot of OPLS-DA model obtained from J and Y samples (POS and NEG).**

(A) Loading Scatter Plot of OPLS-DA model obtained from J and Y (POS). (B) S-plot of OPLS-DA model obtained from J and Y (POS). (C) Loading Scatter Plot of OPLS-DA model obtained from J and Y (NEG). (D S-plot of OPLS-DA model obtained from J and Y (NEG). Ends of the loading Scatter Plot and S-plot of OPLS-DA model were potential differences markers. The farther the distance from the center on behalf of the degree of difference.


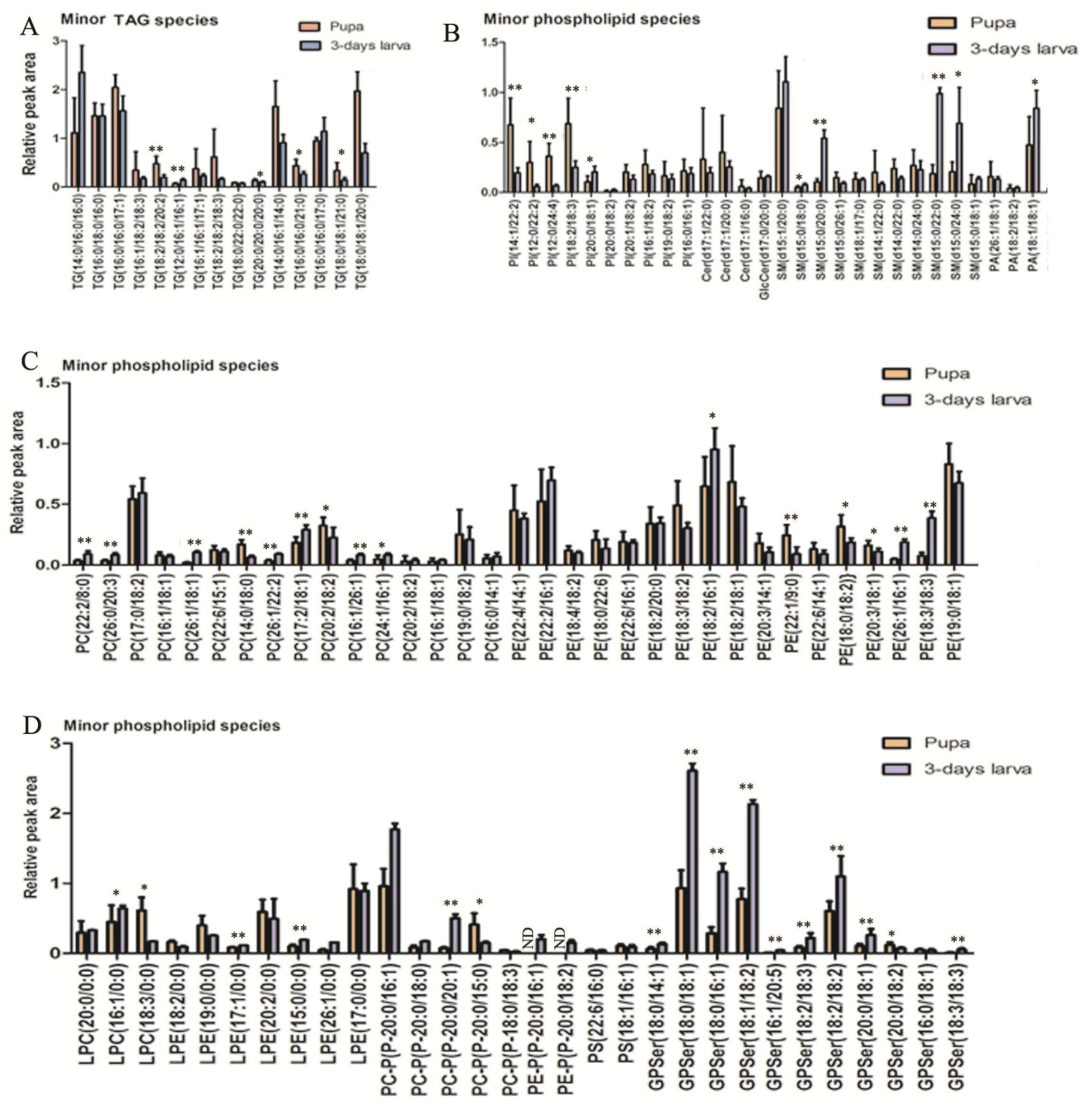


Figure S3. **Changes in lipid composition of PL and TG in 3 days larvae and pupae of** [***L. japonica***](http://www.so.com/link?url=http%3A%2F%2Fwww.doc88.com%2Fp-9611679742241.html&q=%E6%A3%89%E8%9A%9C%E8%8C%A7%E8%9C%82&ts=1463193541&t=8cde705d94909f6d56063ab2923e2fa&src=haosou) ***.***

(A-E) The relative peak area of quantified lipid classes in minor TG (A), middle PL (B) and minor PL (C-E) in 3 days larvae and pupae of [*L. japonica*](http://www.so.com/link?url=http%3A%2F%2Fwww.doc88.com%2Fp-9611679742241.html&q=%E6%A3%89%E8%9A%9C%E8%8C%A7%E8%9C%82&ts=1463193541&t=8cde705d94909f6d56063ab2923e2fa&src=haosou) . Data are presented as means + SEM; n = 6 for both groups. Significance level: *p < 0.05, **0.01<p < 0.05,***p<0.01. N.D., not detected.

Table S2: FPKM (fragments per kilobase per million mapped reads) of selected genes related to figure 6.

| Gene | 3days-1 | 3days-2 | 3days-3 | 3days-3 | Pupa-2 | Pupa-3 | Log_2_FD  (pupa/3days) |
| --- | --- | --- | --- | --- | --- | --- | --- |
| gpi | 14.86 | 23.99 | 24.29 | 30.68 | 27.85 | 30.97 | -0.47 |
| fbpa | 3.51 | 1.65 | 1.08 | 2 | 1.28 | 1.13 | -1.23 |
| tim | 82.51 | 160.27 | 123.15 | 86.56 | 90.73 | 75.67 | -1.44 |
| g6pd | 14.22 | 11.62 | 9.4 | 7.91 | 6.63 | 6.53 | -1.63 |
| gapdh | 14.74 | 2.74 | 1.33 | 0.19 | 0.69 | 0.39 | -3.95 |
| gk | 3.95 | 6.15 | 10.41 | 7.77 | 5.97 | 7.62 | -0.92 |
| agpat1 | 6.57 | 14.64 | 8.54 | 2.95 | 3.26 | 4.7 | -2.34 |
| agpat2 | 3.93 | 7.27 | 10.39 | 2.38 | 2.11 | 1.06 | -2.74 |
| ppap2 | 4.59 | 0.66 | 5.76 | 0 | 0.09 | 0.09 | -5.31 |
| dgat | 12.66 | 22.96 | 14.25 | 0.79 | 0 | 0.22 | -5.78 |
| tgl3 | 12.23 | 8.65 | 22.62 | 1.61 | 1.48 | 2.03 | -3.92 |
| pgam | 91.67 | 101.74 | 74.32 | 72.91 | 65.92 | 80.26 | -1.22 |
| pk | 22.14 | 94.32 | 116.23 | 30.37 | 27.52 | 32.86 | -2.24 |
| cs | 11.38 | 1.71 | 0.36 | 0 | 0 | 0 | -5.89 |
